# Injected Human Muscle Precursor Cells Overexpressing PGC-1*α* Enhance Functional Muscle Regeneration after Trauma

**DOI:** 10.1155/2018/4658503

**Published:** 2018-01-21

**Authors:** Deana Haralampieva, Souzan Salemi, Thomas Betzel, Ivana Dinulovic, Stefanie D. Krämer, Roger Schibli, Tullio Sulser, Christoph Handschin, Simon M. Ametamey, Daniel Eberli

**Affiliations:** ^1^Department of Urology, Laboratory for Tissue Engineering and Stem Cell Therapy, University Hospital Zürich, University of Zürich, Frauenklinikstrasse 10, 8091 Zürich, Switzerland; ^2^Institute for Pharmaceutical Sciences, ETH Zürich, Vladimir-Prelog-Weg 1-5/10, 8093 Zürich, Switzerland; ^3^Zürich Center for Integrative Human Physiology (ZIHP), Zürich, Switzerland; ^4^Biozentrum, Focal Area Growth and Development, University of Basel, Klingelbergstrasse 50-70, 4056 Basel, Switzerland

## Abstract

While many groups demonstrated new muscle tissue formation after muscle precursor cell (MPC) injection, the capacity of these cells to heal muscle damage, for example, sphincter in stress urinary incontinence, in long-term is still limited. Therefore, the first goal of our project was to optimize the functional regenerative potential of hMPC by genetic modification to overexpress human peroxisome proliferator-activated receptor gamma coactivator 1-alpha (hPGC-1*α*), key regulator of exercise-mediated adaptation. Moreover, we aimed at establishing a feasible methodology for noninvasive PET visualization of implanted cells and their microenvironment in muscle crush injury model. PGC-1*α*-bioengineered muscles showed enhanced marker expression for myogenesis (*α*-actinin, MyHC, and Desmin), vascularization (VEGF), neuronal (ACHE), and mitochondrial (COXIV) activity. Consistently, use of hPGC-1*α*_hMPCs produced significantly increased contractile force one to three weeks postinjury. PET imaging showed distinct differences in radiotracer signals ([^18^F]Fallypride and [^11^C]Raclopride (both targeting dopamine 2 receptors (D2R)) and [^64^Cu]NODAGA-RGD (targeting neovascularization)) between GFP_hMPCs and hD2R_hPGC-1*α*_hMPCs. After muscle harvesting, inflammation levels were in parallel to radiotracer uptake amount, with significantly lower uptake in hPGC-1*α* overexpressing samples. In summary, we facilitated early functional muscle tissue regeneration, introducing a novel approach to improve skeletal muscle regeneration. Besides successful tracking of hMPCs in muscle crush injuries, we showed that in high-inflammation areas, the specificity of radioligands might be significantly reduced, addressing a possible bottleneck of neovascularization PET imaging.

## 1. Introduction

A promising treatment option for various muscle-related diseases, for example, stress urinary incontinence, is the use of autologous stem cells for restoration of damaged muscle fibers, for example, in the urinary sphincter. Muscle precursor cells (MPCs) represent the cell population that has been shown to be indispensable for skeletal muscle regeneration [1]. Due to their potential to differentiate into myoblasts and the ability to later form new contractile myofibers, MPCs are being investigated for muscle tissue engineering and reconstruction in the treatment of a variety of muscle diseases [2–4]. Still, two main concerns about their successful and safe clinical application are unsolved: the volumetric loss of the bioengineered tissues over time and the missing tools for noninvasive imaging of the fate of the implanted cells.

A major shortcoming of the implantation of hMPCs is their decreased growth capacity in the aged population [5–7]. This challenge for autologous cell therapy may be addressed by exercise and/or therapeutic regulation of gene expression, which enhances the ability of MPCs to restore muscle fibers. Reduced physical activity is linked to many chronic diseases. The understanding of the molecular mechanisms behind the regeneration of impaired muscle tissue (e.g., damaged urinary sphincter) is vital for the development of proper treatment strategies. One key player in the regulation of exercise-mediated adaptations and of neuromuscular activity of skeletal muscles is the transcriptional coactivator peroxisome proliferator-activated receptor (PPAR) *γ* coactivator 1*α* (PGC-1*α*) [8, 9]. Its expression in muscle tissue is proportional to the amount of exercise and has been shown to counteract atrophy [10]. Atrophy is a major limitation hindering skeletal muscle tissue bioengineering. PGC-1*α* plays an essential role in the regulation of cellular differentiation, development, and metabolism (carbohydrate, lipids, and protein) of higher organisms [10, 11]. PGC-1*α* is further regulating the mitochondrial biogenesis and is adapting the oxidative state in muscles. In skeletal muscle, PGC-1*α* is abundant and particularly enriched in slow-twitch, oxidative muscle fibers, containing a large number of mitochondria. Importantly, oxidative muscle fibers are the dominant type in the external urinary sphincter [12]. It has been shown that increased levels of PGC-1*α* can promote a shift in the fiber composition toward high-endurance muscle fibers [13, 14]. Furthermore, this muscle phenotype is characterized by pronounced tissue vascularization [15], increased myoglobin levels, and enhanced import of glucose, lipids, and lactate [16]. In addition, PGC-1*α* tightly links muscle and nerve by regulating neuromuscular junction genes and by promoting clustering of acetylcholine receptors (ACHR) at the motor endplate [17]. It has been proposed that PGC-1*α* controls muscle plasticity and suppresses a broad inflammatory response [18]. There is further evidence suggesting that oxidative metabolism and inflammation counteract each other in muscle tissue, outlining the central function of PGC-1 *α* in muscle recovery. Therefore, emphasizing the use of PGC-1*α* alteration opens up for novel muscle tissue engineering approaches [19] and possible clinical applications [20] in urology and beyond.

Besides the volumetric loss of bioengineered tissues, another shortcoming in the field is the missing tools for noninvasive imaging of this cell therapy. Research towards the noninvasive imaging of autologous stem cell therapies is of high importance as both repeated biopsy and access to many organs are often not clinically feasible. Several modalities are being investigated for their applicability in cell tracking and cell metabolism read-outs [21]. While MRI has lower sensitivity compared to radionuclide-based tools and bioluminescence has poor spatial resolution, PET/CT is a system with both high sensitivity and resolution [21]. Additionally, although imaging reporter genes are available for fluorescence, bioluminescence, and MRI, only radionuclide-based reporter genes are currently investigated for use in patients [22–27]. In our previous work, we developed a feasible method for *in vivo* tracking of subcutaneously injected hMPCs using [^18^F]Fallypride, a well-established dopamine 2 receptor (hD2R) PET ligand [28]. After the successful generation of adenoviruses for the overexpression of a signalling-deficient hD2R in hMPCs, we were able to establish an ex situ model for imaging of bioengineered muscle tissue [28]. This encouraged us to concentrate our further investigations towards the applicability of these methods in an in situ muscle crush model studying skeletal muscle regeneration, thereby coming closer to a model for studying urinary sphincter muscle restoration.

In this regard, we aimed at examining the influence of genetically modified hPGC-1*α* overexpressing hMPCs, injected in a skeletal muscle after crush injury, specifically investigating the effect on tissue regeneration and muscle contractility. We expected to improve the cellular therapy for clinical implementation in stress urinary incontinence patients in the future, restoring the functionality of the external urinary sphincter (predominantly oxidative type fibers [12]). Moreover, we used a method for noninvasive PET tracking by ectopic hD2R expression in the implanted cells [28] and visualization of the neovascularization in the regenerating muscle tissue. We hypothesize that by enhancing the hPGC-1*α* expression in hMPCs, we can improve the regeneration capacity and contractility of the injured muscles.

## 2. Materials and Methods

### 2.1. Isolation and Expansion of hMPCs

Human muscle biopsies from the *M. rectus abdominis* were collected upon ethical approval and with informed consent of 6 hospitalized patients undergoing abdominal surgery under general anesthesia. The patients were selected according to strict inclusion and exclusion criteria, assuring the optimal quality of the muscle biopsy (e.g., no muscular dystrophy, hormonal therapy, and chronic infectious diseases). The samples were processed according to established protocols [29]. Briefly, each muscle biopsy was first minced and digested with collagenase type I 0.2% (*w*/*v*) (Sigma) and dispase 0.4% (*w*/*v*) (Gibco). The enzymatic reaction was terminated with medium containing 10% FBS. Individual fibers were then liberated by rigorous pipetting and filtered through a strainer with a pore size of 100 *μ*m. After centrifugation, the pellet was resuspended in culture medium and the muscle fibers transferred into 35 mm dishes coated with collagen type I (1 mg/ml) (BD). The culture medium consisted of DMEM/F12, 1% penicillin/streptomycin, 18% FBS, 10 ng/ml hEGF (Sigma), 1 ng/ml hbFGF (Sigma), 10 *μ*g/ml human insulin (Sigma), and 0.4 *μ*g/ml dexamethasone (Sigma) [29]. After 24 h, a fibroblast reduction step was performed by replating the cells. The cultured hMPCs were characterized as published before [14, 29].

### 2.2. Adenoviral Design and Transduction

The AdEasy System was used as a tool for recombinant adenovirus generation. For the first construct, N-terminal HA-tagged human PGC-1*α* was cloned into an adenoviral vector that codes for CMV promoter-driven green fluorescent protein (GFP). The expression of hPGC-1*α* was also under the control of a CMV promoter, thereby ensuring its robust, constitutive expression [14]. For the second construct, phenylalanine 411 of the human D2R was mutated into alanine (F411A) to obtain a signalling-deficient human dopamine D2 receptor that still binds ligands in a normal manner but will not activate intracellular signalling upon ligand binding [28, 30]. As a control for viral infection, a GFP adenovirus was used. The viral titer was increased through additional amplification steps and quantified by fluorescent microscopy. The optimal multiplicity of infection (MOI) was measured by serial titrations of the viral vectors on hMPCs and simultaneous determination of fluorescent cells, cell toxicity, and cell viability and proliferation. Detailed descriptions of the performed assays were published before [14, 28]. Finally, the transduced hMPCs were expanded for 2 days after infection and were injected at the injured site in nude mice.

### 2.3. Animal Experimentation

All animal experiments were approved by and performed according to the local commission for animal experiments. A total of 49 female 8-week-old, nude mice (Charles River, Germany) received hind limb lateral incisions on both sides (from the inferior tibiofibular joint up to the knee joint) under general anesthesia (3% isoflurane) and aseptic conditions, according to a modified published protocol [31]. Briefly, a coronal plane beneath the *tibialis anterior* (TA) was opened, separating the muscle from the tibia. The lower jaw of nonserrated forceps was gently inserted below the TA. Crush injury was performed by closing the forceps to its first stage for 3 seconds. The forceps was gently removed, the hMPC collagen suspension was injected, and the wound was closed. Each injection contained 6 × 10^6^ transduced hMPCs, which were gently mixed with 100 *μ*l collagen type I carrier (final concentration: 2 mg/ml) (BD) prior to injection. A volume of 30 *μ*l of the collagen cell suspension could be injected without leakage. The muscles were harvested 9 ± 1 days (early), 18 ± 3 days (midterm), or 31 ± 3 days (late) after injection. The deviation in time periods was mandatory due to a complex regimen in repetitive PET imaging with different tracers in the same animal.

### 2.4. Radiosynthesis of [^11^C]Raclopride

The radiosynthesis of [^11^C]Raclopride was successfully accomplished using an established procedure in our lab. Briefly, cyclotron-produced [^11^C]CO_2_ gas was reacted with H_2_ using Ni catalyst to afford [^11^C]CH_4_, which was passed through an I_2_ column to yield [^11^C]CH_3_I. [^11^C]CH_3_I was then reacted with the desmethyl precursor for 5 min at 90°C. After HPLC purification and SPE extraction, [^11^C]Raclopride was obtained in 99% radiochemical purity with a maximal specific activity of 239 GBq/*μ*mol. A total of 1.09–1.72 GBq of [^11^C]Raclopride was obtained in an injectable solution of 5% EtOH in 0.15 M PBS. PET scans with [^11^C]Raclopride were acquired from 0 to 60 min p.i., and time frames were averaged for data analysis.

### 2.5. Radiosynthesis of [^64^Cu]NODAGA-RGD

In order to image neovascularization, we used [^64^Cu]NODAGA-RGD to target the growth factor integrin *α*_v_*β*_III_. Ammonium ascorbate (0.5 M, pH 5.5) was added to [^64^Cu]CuCl_2_ (50 *μ*l in 0.05 N HCl) obtained commercially or produced at Paul Scherrer Institute (PSI), followed by an addition of 40 *μ*l of NODAGA peptide (NODAGAc(RGDfk), 1 mM in H_2_O). Labeling was carried out at 95°C for 15 minutes. According to analytical HPLC, 90% of ^64^Cu activity was chelated and only 10% [^64^Cu]CuCl_2_ remained unreacted. To bind this remaining fraction, 5 *μ*l DTPA (1 g/15 ml) was added. The resulting [^64^Cu]DTPA complex is known to be easily excreted through the renal pathway. Animals were injected with 5–10 MBq [^64^Cu]NODAGA-RGD in the tail vein and were imaged 17–22 h p.i.

### 2.6. Standardized Uptake Value (SUV)

SUV is a semiquantitative parameter representing radioactivity concentration in tissue. It is mathematically defined as the ratio of tissue radioactivity concentration to injected radioactivity per kilogram body weight at a certain point during the PET studies.

### 2.7. Immunohistological Assessment

The harvested TA muscles were embedded in cryopreservative (OCT embedding medium, Cell Path) immediately after isolation. Cryostat sections were prepared (10 *μ*m) and further processed. For immunohistological analysis, the tissues were fixed (4% PFA, 10 min), permeabilized (0.5% TritonX-100, 20 min), blocked for 30 min (5% BSA+ 0.1% TritonX-100 in PBS), and finally stained with anti-sarcomeric *α*-actinin (1 : 200, Sigma) and F4/80 (1 : 100, Abcam) over night at 4°C. After washing with PBS, the tissues were incubated with Cy3 anti-mouse IgG secondary antibody (1 : 1000, Sigma) and DAPI (1 : 100, Sigma) for 1 h at room temperature, washed again, and finally mounted (Dako). Images were acquired with a Leica-Imager Type DM6000B at exposures normalized to unstained controls (secondary antibody and DAPI only).

### 2.8. Macrophage Staining Analysis

A computer-assisted approach was used to quantify macrophage (F4/80) immunolabelling. Longitudinal TA muscle sections were imaged using the Leica-Imager Type DM6000B, and the images used for analysis were captured from the crushed (central) region of the harvested muscles. For evaluation of the signal (% area), 5–20 high-power fields (HPF, 20x) were analyzed by ImageJ per time point and per group.

### 2.9. Real-Time PCR

For the analysis of PGC-1*α* downstream-regulated genes and skeletal contractile muscle genes in the regenerating tissue by RTPCR, the middle of the crushed region of each harvested TA muscle was excised, pulverized in liquid nitrogen, and suspended in RNA lysis buffer. Total RNA was isolated using the SV Total RNA Isolation System kit (Promega) according to the manufacturer's protocol, including a DNase digestion. RNA was reverse transcribed with random primers (high-capacity cDNA reverse transcription, Life Technologies). Predesigned primers for human PPARGC-1 (Hs01016719_m1), D2DR (dopamine 2 receptor, Hs00241436_m1), VEGF (vascular endothelial growth factor, Hs00900055_m1), MyH1 (myosin heavy chain 1, Hs00428600_m1), MyH2 (myosin heavy chain 2, Hs00430042_m1), Desmin (Hs00157258_m1), *α*-actinin (Hs00998100_m1), COXIV (cytochrome c oxidase subunit 4,Hs00971639_m1), vWf (Mm00550375_m1), TNF-*α* (tumor necrosis factor alpha, Mm00443258_m1), and ACHE (acetyl choline esterase, Hs00241307_m1) were purchased from Life Technologies. 18S rRNA (4319413E) was used to normalize cDNA concentrations. For quantification, the expression of each gene was normalized to the 18S expression in the corresponding sample.

### 2.10. Organ Bath (Myography)

The muscles were isolated at different time points after the lesion, and the posttraumatic functional recovery was quantitatively assessed by organ bath. After harvesting, the tissues were kept under tension with constant oxygenation (95% O_2_ and 5% CO_2_) in Krebs solution at 25°C. TA muscles were fastened with vicryl into the myograph chambers (DMT, Denmark) and allowed to equilibrate under 15 mN (1.5 g) for 20 min, adjusting the tension periodically and replacing Krebs solution every 5 min. The samples were stimulated by electrical field stimulation (EFS) (80 V, 80 Hz), and 3 measurements per sample were considered for the analysis. Native TA muscle (TA nat) was used as control, and the contraction force was set to 100%. Forces of injured muscles were calculated relatively as the percentage of maximum. The maximum tension under tetanic contraction was registered and normalized to the sample weight (mg/mg tissue). All data were collected using a LabChart v7.0 (AD instruments, Spechbach, Germany) and expressed as mean ± S.E.M.

### 2.11. Statistics

For statistical analysis, IBM SPSS v22.0 (SPSS Inc.) was used and graphics were drawn with GraphPad Prism v5.04 (GraphPad Software Inc.). All data were analyzed by Student's *t*-tests, Mann Whitney *U* test, or one-way ANOVA with Bonferroni or LSD post hoc analysis (*p* < 0.05 was considered significant). All presented data are expressed as means with a corresponding standard error of the mean (±SEM).

## 3. Results

### 3.1. PGC-1*α* Overexpressing hMPCs Enhance the Levels of Contractile Muscle Markers during Tissue Regeneration

**Figure 1:**
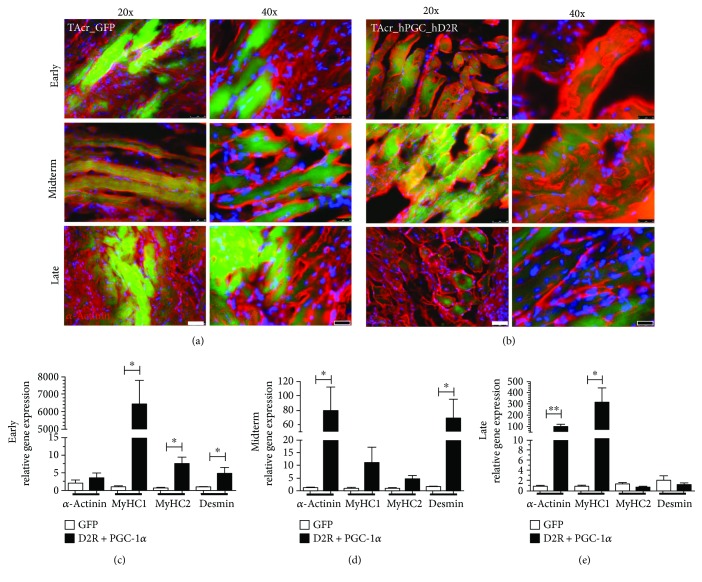
PGC-1*α* induces upregulation of the expression of contractile genes in regenerating muscle. Immunohistological assessment of newly built muscle fibers (green), stained with sarcomeric *α*-actinin antibody (Cy3, red) over time ((a) GFP and (b) PGC-1*α*). RTPCR analysis showed enhanced *α*-actinin, MyHC1, MyHC2, and Desmin gene expression when PGC-1*α* was overexpressed at (c) early (9 ± 1 d), (d) midterm (18 ± 3 d), and (e) late (31 ± 3 d) time points after the TA crush injury. TAcr: *tibialis anterior* crushed. ^∗^*p* < 0.05 and ^∗∗^*p* < 0.01.

After successful isolation, expansion, and characterization of hMPC from six patient biopsies [14] [28], we evaluated the effects of PGC-1*α* overexpressing hMPCs in a TA crush injury model. To directly assess the role of PGC-1*α* in myofiber formation, immunofluorescent microscopy and RTPCR analysis of the regenerating crushed muscle tissue were performed (Figure 1). The participation of transduced GFP-positive cells in the myotube formation (*α*-actinin, Cy3) over time could be visualized by fluorescent microscopy in GFP-infected samples (Figure 1(a)) and in PGC-1*α*-infected samples (Figure 1(b)). In line with our previous ex situ results [14], PGC-1*α* overexpressing-engineered muscle tissue showed increased relative expression also at gene level for sarcomeric *α*-actinin (3.47 ± 1.45, *n* = 6, *p* = 0.5476), MyHC1 (6440.64 ± 1370.88, *n* = 6, *p* = 0.0238), MyHC2 (7.51 ± 2.03, *n* = 6, *p* = 0.0238), and Desmin (4.78 ± 1.76, *n* = 6, *p* = 0.0476) at early time points in the regenerating tissue, relative to GFP samples (Figure 1(c)).

While the Desmin and MyHC2 gene expression reached equivalent levels in PGC-1*α* and GFP samples over time (Figure 1(e); 1.28 ± 0.29, *n* = 12, *p* = 0.5425; 0.55 ± 0.18, *n* = 18, *p* = 0.0546), the expression of *α*-actinin and MyHC1 remained significantly higher in PGC-1*α* samples at late time points (Figure 1(e); 96.15 ± 24.43, *n* = 15, *p* = 0.0092; 312.22 ± 132.8, *n* = 18, *p* = 0.015), suggesting a hPGC-1*α*-induced shift towards slow-twitch type fibers.

### 3.2. Increased Muscle Contraction through PGC-1*α* Overexpressing hMPC Injection after Crush Injury

**Figure 2:**
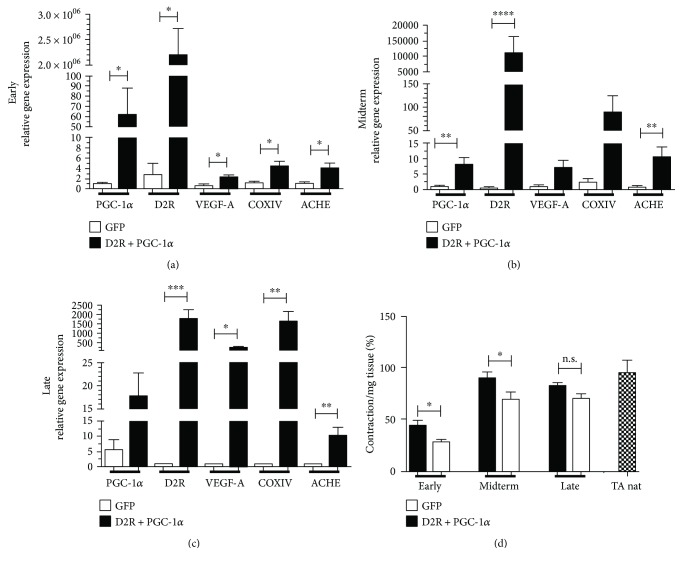
PGC-1*α*_hMPCs induce expression of genes related to vascularization, mitochondrial, and neuronal activation and enhance the contractility at early and midterm time points after TA crush injury during regeneration. RTPCR analysis confirmed the sustained overexpression of hPGC-1*α* and the signalling-deficient hD2R genes at the crush injury site over time. Relative VEGF-A, COXIV, and ACHE gene levels were enhanced in the corresponding samples, compared to control GFP_hMPC at (a) early, (b) midterm, and (c) late time points of regeneration. (d) PGC-1*α* overexpression led to increased TA contractile force at early and midterm time points. Native TA muscle (TA nat) was used as control, and the contraction force was set to 100%. Forces of injured muscles were calculated relatively as the percentage of maximum. VEGF-A: vascular endothelial growth factor-a, COXIV: cytochrome c oxidase subunit 4, ACHE: acetylcholine esterase. ^∗^*p* < 0.05, ^∗∗^*p* < 0.01, ^∗∗∗^*p* < 0.001, and ^∗∗∗∗^*p* < 0.0001.

Encouraged by the enhanced gene expression of contractile markers in the regenerating muscles (Figure 1) with increased PGC-1*α* levels, we decided to analyze the expression of further markers, known to be involved in muscle regeneration. The crushed/regenerating tissue was investigated by RTPCR for the expression of factors connected to vascularization (VEGF-A), mitochondrial activity (COXIV), and neuronal activity (ACHE). The overexpression of hD2R and hPGC-1*α* was sustained over time (Figures 2(a)–2(c)). Notably, the PGC-1*α* “native” expression in GFP-infected samples increased at the latest time point (Figure 2(c)). Increased levels of VEGF-A in hPGC-1*α* overexpressing samples, relative to GFP (Figures 2(a)–2(c)), were detected at early (8–10 d) (A: 2.26 ± 0.49, *n* = 6, *p* = 0.0476), midterm (14–21 d) (B: 7.18 ± 2.46, *n* = 21, *p* = 0.1475), and late (28–35 d) (C: 204.65 ± 49.7, *n* = 18, *p* = 0.0124) time points. Similarly, the PGC-1*α* downstream-regulated mitochondrial activity was enhanced (COXIV) (A: 4.32 ± 1.02, *n* = 6, *p* = 0.0238; B: 88.87 ± 38.06, *n* = 21, *p* = 0.1475; and C: 1595.35 ± 547.55, *n* = 18, *p* = 0.003). Finally, the gene expression levels of ACHE illustrated the expected increase in the PGC-1*α*-modified regenerating tissues (A: 3.96 ± 1.04, *n* = 6, *p* = 0.0238; B: 10.52 ± 3.51, *n* = 18, *p* = 0.0012; and C: 9.99 ± 2.89, *n* = 12, *p* = 0.0032). In line with the enhanced gene expression of markers for contractility, vascularization, and neurons, we observed an increased muscle contraction in hPGC-1*α* overexpressing muscles. Organ bath measurement at 80 V and 80 Hz revealed significantly elevated contraction force at early (PGC-1*α*: 43.75 ± 5.27, *n* = 6; GFP: 28.36 ± 2.89, *n* = 13, *p* = 0.0337) and midterm (PGC-1*α*: 89.49 ± 6.14, *n* = 33; GFP: 69.85 ± 7.12, *n* = 18, *p* = 0.0435) time points in hPGC-1*α*-treated muscles (Figure 2(d)). At late time points, both hPGC-1*α* and GFP overexpressing muscles contracted at similar levels (PGC-1*α*: 82.33 ± 3.73, *n* = 21; GFP: 70.52 ± 5.17, *n* = 9, *p* = 0.0830) as TA native control. All measurements are relative to contraction of TA native, which was set as 100%.

### 3.3. Tracking of hMPC in a TA Muscle Crush Injury by PET/CT

**Figure 3:**
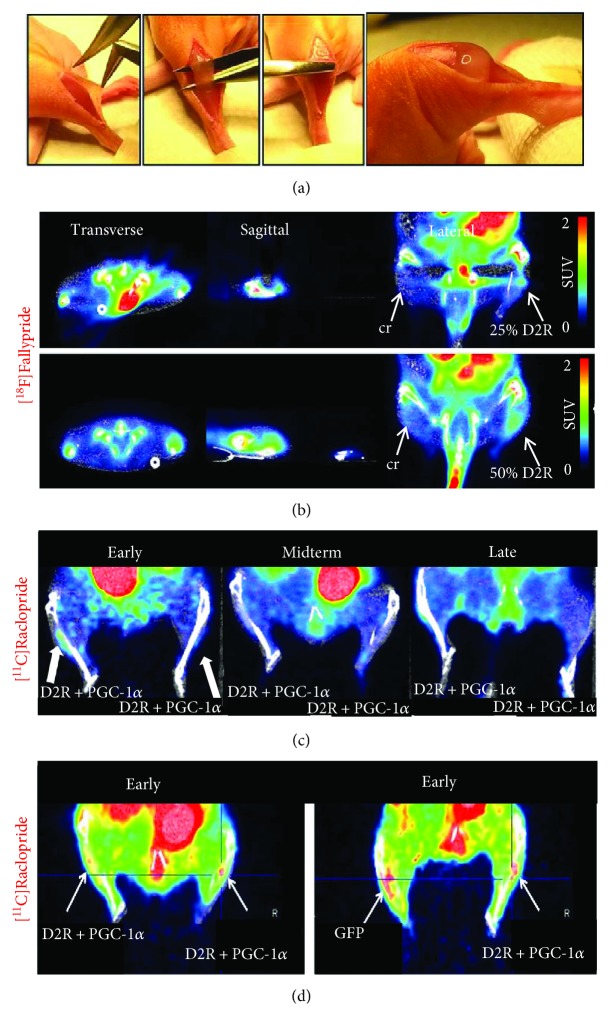
PET/CT tracking of hD2R_hMPCs in a TA crush injury model. (a) Nude mice were subjected to a TA crush injury, followed by injection of cell collagen suspension. (b) Feasible tracking of hD2R_hMPCs with [^18^F]Fallypride in a virus dose-dependent manner (25% versus 50% hD2R-positive cells). No tracer uptake in the TA crush only (cr). (c) [^11^C]Raclopride accumulation in hD2R_hPGC-1*α*_hMPCs at early time points, but not at midterm and late. (d) Enhanced accumulation of [^11^C]Raclopride at GFP_hMPC injection site, compared to hD2R_hPGC-1*α*_hMPCs. SUV: standardized uptake value (the same scale used for all), cr: crush only.

A crush injury was introduced to the TA muscle of nude mice (Figure 3(a)). The injected hD2R_hMPCs were successfully tracked in the crush injury region using the specific D2R radiotracer [^18^F]Fallypride (early time point), resulting in a virus dose-dependent signal (Figure 3(b)). The injection of 50% hD2R-positive cells in the damaged tissue led to an increased radiotracer uptake, compared to the injection of 25% positive cells. No signal could be detected in the crush only controls. To exclude signal contamination from ^18^F-fluoride in the tibial bone, [^11^C]Raclopride was used as an alternative high-affinity D2R ligand for further experiments. For these experiments, a viral combination of hD2R and hPGC-1*α*, compared to GFP control infected cells, was used (Figures 3(c) and 3(d)). Uptake of [^11^C]Raclopride could be detected in hD2R_hPGC-1*α*_hMPCs at early time points only (Figure 3(c)). Further analysis indicated an unspecific radiotracer uptake at the site of injury also when GFP_hMPCs were used (Figure 3(d)). Importantly, the accumulation of the radiotracer in the intestines, urinary bladder, and joints (18F–fluoride) is a known PK (pharmacokinetic) property of the radiotracer and should be considered physiologic and nonspecific accumulation for the current application.

### 3.4. PGC-1*α* Overexpressing hMPCs Reduce the Proinflammatory Response after Muscle Crush Injury

**Figure 4:**
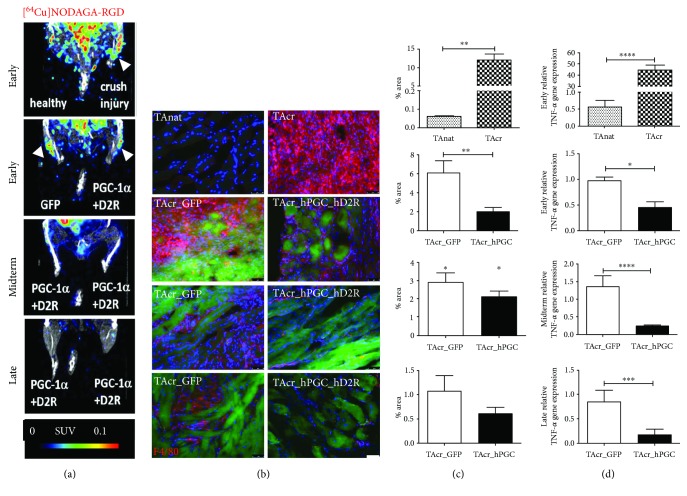
PGC-1*α* overexpression in hMPCs reduces the inflammatory response in the crush injury. (a) Unspecific accumulation of neovascularization radiotracer [^64^Cu]NODAGA-RGD in the early time points after the injury. (b) Immunohistological assessment of macrophage (F4/80, red) invasion in the injured region with and without hMPCs (green). (c) Evaluation of the fluorescent intensity showed increased F4/80 signal in TA crush only, compared to TA native (early), and reduced signal at the site of injection of hD2R_hPGC-1*α*-infected cells, compared to GFP (early). The latter trend was visible also at later time points (midterm, late). (d) RTPCR analysis of the relative TNF-*α* gene expression in the crushed tissue could further confirm the anti-inflammatory effect of PGC-1*α* overexpressing hMPCs, sustained over time. SUV: standardized uptake value, TAnat: *tibialis anterior* native, TAcr: *tibialis anterior* crushed. ^∗^*p* < 0.05, ^∗∗^*p* < 0.01, ^∗∗∗^*p* < 0.001, and ^∗∗∗∗^*p* < 0.0001.

Initially aiming at imaging of neoangiogenesis with the specific *α*_v_ß_III_ radiotracer [^64^Cu]NODAGA-RGD, we observed a highly increased accumulation of the tracer in TA crush only in the early periods after injury, when compared to native TA and to the crushed muscles with injected hMPCs (Figure 4(a), early). At later time points, no PET signal was detectable (Figure 4(a), midterm and late). Furthermore, immunohistological analysis of harvested muscles with a macrophage expression marker (F4/80, Cy3) demonstrated (1) a highly increased macrophage accumulation in crushed TA compared to native (Figures 4(b) and 4(c), early) and (2) a decreased signal in the hD2R_hPGC_hMPCs compared to GFP_hMPCs (Figures 4(b) and 4(c), early). The latter effect was observed also at later time points (Figures 4(b) and 4(c), midterm and late). RTPCR analysis of the proinflammatory cytokine TNF-*α* in the crushed/regenerating tissue confirmed the highly increased signal in TA crush versus TA native (Figure 4(d), early, 44.73 ± 4.52, *n* = 3, 0.57 ± 0.19, *n* = 6, *p* < 0.0001) and the decreased expression in PGC-1*α* overexpressing tissues compared to GFP (Figure 4(d), early, 0.443 ± 0.11, *n* = 3, 0.98 ± 0.07, *n* = 3, *p* = 0.0177). The PGC-1*α*-related reduction of inflammation was sustained also at midterm (Figure 4(d), 0.23 ± 0.03, *n* = 15, 1.35 ± 0.30, *n* = 3, *p* < 0.0001) and late time points (Figure 4(d), 0.15 ± 0.02, *n* = 9, 0.84 ± 0.24, *n* = 3, *p* = 0.0005).

## 4. Discussion

Autologous stem cell therapy is on the doorstep to successful clinical application and represents a novel treatment option for various muscle-related pathologies, including urinary and anal incontinence, vocal cord dysfunction, and reflux. Muscle precursor cells, or activated satellite cells, are responsible for the regeneration in postnatal skeletal muscles. In the past decades, these cells have been investigated for muscle tissue bioengineering approaches, allowing the growth of new myofibers [2, 3, 32, 33]. However, there are certain limitations in the quality of the de novo engineered constructs. The process of proliferation and differentiation of these cells is largely driven by growth factors and altered by tissue injury or exercise [34, 35]. To address this matter, we designed a model for facilitated muscle regeneration after injury, by inducing overexpression of hPGC-1*α* in the injected hMPCs in the injured skeletal muscles.

Intensive research has shown that the natural process after muscle injury follows a highly conserved sequence of steps, leading to the restoration of tissue architecture, and importantly also function [36]. A crucial step in the process of forming new muscle tissue is the capacity of MPCs to differentiate into myotubes. Consistent with the previously reported facilitated *in vitro* and *in vivo* ex situ differentiation of hPGC-1*α*_hMPCs [14], the regenerating TA muscles with hPGC-1*α* overexpression demonstrated earlier myotube formation in situ. This would facilitate the functional regeneration in patients with a damaged urinary sphincter. The injection of hPGC-1*α*_hMPC in the crush injury also significantly increased the expression of contractile proteins at all time points after the injury, indicating facilitated restoration compared to GFP-infected controls. Importantly, the expression of MyHC1 was significantly increased in the hPGC-1*α*_hMPC samples, correlating with our goal to bioengineer predominantly slow-twitch muscle fibres, such as in the external urinary sphincter [12]. In line with our *in vivo* observations, others have shown that an increased VEGF release in a hypoxic environment leads to enhanced differentiation of muscle cells [37]. It has been demonstrated that secretion of various effectors by injected hMPCs contribute to optimization of the regenerative process (e.g., neovascularization) [32, 38, 39]. The hPGC-1*α*-related regeneration enhancement was further promoted by an increase in factors for mitochondrial (COXIV) and neuromuscular (ACHE) activity, which are known to be vital for successful muscle tissue bioengineering. Further supporting evidence suggested an increased expression of mitochondrial and other metabolic genes as a plausible mechanism for rescuing a damaged muscle [18]. All these factors support the observed facilitated contraction force production by the regenerating muscles injected with hPCG-1*α*_hMPC. At the later time points, there was no significant difference between the contraction forces of the two groups. This was expected, as the regeneration of the muscles at later stages was completed, and there was no difference to native TA contraction. Although the functional results (organ bath) suggest a diminished difference between the two groups in long-term, the data support our main aim for designing a model for significantly facilitated muscle regeneration. One has to consider certain limitations of the approach; for example, the use of young mice brings along fast self-regeneration that might not be the best comparison model to the real situation in elderly patients. Nevertheless, we could show significant improvement of the regeneration speed even in a highly self-regenerative environment. We expect to observe an even more prominent effect on facilitated muscle regeneration in patients. Moreover, GFP_hMPC-injected muscles showed increased PGC-1*α* gene expression levels at the latest stages, comparable to these in hPGC-1a_hMPC samples.

While resistance training combined with adequate nutrition remains the most effective intervention to diminish the functional decline in muscles, there is a certain age-linked barrier to obtaining full benefits from this therapy [40]. PGC-1*α* would be a promising “exercise molecule,” which controls skeletal muscle metabolism and has potential therapeutic effects. Therefore, we believe that by inducing hPGC-1*α* overexpression in the injected hMPCs, we are able to mediate muscle-healing effects for structural, metabolic, and functional restoration, irrespective of physical status and age of the patient. This makes the current preclinical approach a good candidate for regenerating the urinary sphincter in patients suffering from stress urinary incontinence in near future.

Besides improving the quality of the engineered skeletal tissues, we aimed at establishing a method to noninvasively image the cell fate after implantation, circumventing the need for a tissue biopsy. Molecular imaging with PET is gaining increasing importance in regenerative medicine due to the possibility for noninvasive metabolic read-outs. A recent study described the feasibility of imaging human Na/I symporter (NIS) expression in two mouse models of muscular dystrophy and vascular disease by bioluminescence and PET imaging [41]. This study demonstrated that luciferase- and NIS-engineered skeletal muscle could successfully be imaged. In our present study, we presented a method for noninvasive visualization of the implanted cells in the TA crush injury using PET/CT imaging of hD2R. Tracking of hMPCs via ectopic expression of a signalling-deficient hD2R was previously reported in an ex situ muscle tissue formation model [28]. We were able to visualize the cells in a viral dose-dependent manner in the injured TA muscles of the animals using the highly-specific hD2R radiotracer [^18^F]Fallypride. Nevertheless, the proximity of the muscle injury to the tibial bone led to unspecific signal uptake due to possible defluorination of the radiotracer. To circumvent this problem, we used [^11^C]Raclopride as an alternative hD2R PET imaging agent.

Interestingly, animals injected with GFP-only hMPCs showed higher [^11^C]Raclopride uptake, compared to the regenerating muscles with injected hD2R_hPGC-1*α*_hMPCs. This was also true for other tracers investigated in the study (e.g., [^18^F]FDG, data not shown). Additionally, while aiming at visualizing neovascularization using [^64^Cu]NODAGA-RGD, a relatively high amount of radioactivity was detected in the TA crushed only, where no cells were injected. These observations led to the assumption that the recorded signals were rather related to inflammation, than to vascularization or specific hD2R detection. Immunohistological analysis with the macrophage marker F4/80 and the TNF-*α* gene expression levels in the harvested samples correlated with [^64^Cu]NODAGA-RGD tracer uptake in the injury region. In line with our observations, another study revealed that in addition to neovessels and myofibroblasts, macrophages have also been shown to express *α*_v_*β*_III_ integrin [42]. However, the relative amounts of integrin in these cell types have not been followed over time. Importantly, we were able to show that hPGC-1*α* overexpression in the hMPCs could significantly reduce proinflammatory cytokine expression (TNF-*α*) and enhance the healing process. A relation between hPGC-1*α* and suppression of the broad inflammatory response has previously been reported [18].

## 5. Conclusions

Based on the findings above, hPGC-1*α* overexpressing hMPCs hold a promise for the enhanced repair of skeletal muscle tissue. They demonstrated capacities to amplify the expression of contractile markers, to facilitate the contraction force generation in the regenerating muscles, and to decrease the inflammatory response after crush injury. Additionally, we were able to track the implanted cells in the crushed muscles using PET radioligands. Nonetheless, several challenges remain which need to be overcome in order to establish a feasible method for metabolic imaging of this cellular therapy.
